# A case report of one vasovagal syncope patient with third-degree atrioventricular block caused by SCN5A gene mutation and literature review

**DOI:** 10.1186/s12887-020-02123-8

**Published:** 2020-05-12

**Authors:** Lu Gao, Xia Yu, Hongxia Li, Yue Yuan

**Affiliations:** Department of Cardiology, Beijing Children’s Hospital, Capital Medical University, National Center for Children’s Health, Beijing, China

**Keywords:** Children, SCN5A, Vasovagal syncope, Vagal ganglion modified ablation, Third-degree atrioventricular block

## Abstract

**Background:**

Vasovagal syncope (VVS) is common in children and significantly affects their quality of life. To our knowledge, this the first case report of SCN5A gene mutation associated with VVS and third-degree atrioventricular block (atrioventricular block, AVB), which could help pediatricians aware that VVS is not always a benign condition and help to identify VVS children at the risk of sudden death.

**Case presentation:**

A twelve-year-old male child was admitted to Beijing Children’s Hospital of Capital Medical University for chest tightness for 9 days and syncope in July 2018. The child was diagnosed as VVS with third-degree AVB after complete investagations. A heterozygous mutation in the exon coding region of the SCN5A gene, C. 5851G > T (coding region 5551 nucleotide changed from G to T), was detected in the peripheral blood of the child. Electrophysiological examination and modified vagal ganglion radiofrequency ablation were performed in the child. The ECG playback was normal on the second day after operation. Holter showed no abnormality and no chest tightness or syncope occurred after 3 months and 1 year follow-up.

**Conclusions:**

Our case report firstly reported that SCN5A mutation contributed to the pathogenesis of VVS with third-degree AVB. Vagal ganglion modified ablation have obtained good therapeutic effect. Gene analysis was of great value to the accurate diagnosis and treatment of VVS children.

## Key notes

- Vasovagal syncope is not always a benign prognosis.
- Various aspects were invovled in the pathogesis of vasovagal syncope.
- SCN5A played an important role in vasovagal syncope.

## Background

Vasovagal syncope (vasovagal syncope, VVS) is a common inducement of syncope in childhood owing to a transient decrease of cerebral blood flow which could be caused by a wide variety of predispositions. Vasovagal syncope (VVS) accounting for 60–80% of cases of neurally mediated syncope, is the most common type of autonomic nerve-mediated syncope [1]. VVS results from acute orthostatic intolerance and recurrent syncope seriously affecting the daily life and learning quality of children. Furthermore, cardiac inhibition induced by intense vagal reflex could be observed in most severe syncope cases. What’s more serious is that some children even are at the risk of sudden death [2]. In addition to autonomic nerve-mediated syncope, cardiogenic syncope is also an important cause of syncope in children. Atrioventricular block (AVB) could cause complete atrioventricular segregation because of abnormal conduction of a part of the atrioventricular conduction system [3] which can cause syncope or even sudden death [4]. There are many reasons for the occurrence of third-degree AVB, most of which are secondary. The age of onset was almost over 50 years [4]. Previous reports suggested that VVS and Brugada syndrome showed some relation [5–7].

Previous studies discovered that gene mutation of SCN5A showed correlation with long QT syndrome, Brugada syndrome, atrioventricular block and etc. Nevertheless, the gene mutation of SCN5A in VVS children with third-degree AVB is not well-known. We reported a child with VVS and third-degree AVB of gene mutation of SCN5A and reviewed the current literature.

## Case presentation

A twelve-year-old male patient was admitted to Beijing Children’s Hospital of Capital Medical University for chest tightness for 9 days and syncope in July 2018. There was a history of scarlet fever 2 months ago. There were denying history of trauma, transfusion, food or drug allergy and poisoning. The child had a history of carsickness. He experienced syncope during carsickness occurrence, vomiting and sweating before the syncope attack with lips pale. He was unconscious for approximately 1 min. When he recovered to consciousness, his limbs were weak and pale. The child did not have special birth history with normal growth and development. The mother of the child was healthy. While the father had a history of VVS. No protrusion in the precordial region, no diffuse heart beats, no tremor or pericardial friction, no cardiac boundaries were displayed in the physical examination. No heart murmur sound was heard in the auscultation area of each valve. 24-h dynamic electrocardiogram showed that P-P interval and RR interval had their own fixed rules, P wave and QRS wave had no fixed relationship, atrial rate had no fixed relationship with ventricular rate, by which third-degree AVB was diagnosed. Holter records showed ventricular arrest in the child during carsickness when he was attacked by syncope. The child was diagnosed as VVS according to the results of DC and head-up tilt test (head-up tilt test, HUTT). And ECG, echocardiography, MR scan and EEG were normal. A few mintues after the child standing on the tilt bed in HUTT, bradycardia was observed accompanied with syncope aura, such as pale, sweating and weakness. The child recovered after the tilt-bed returned to recumbent position. Laboratory examination: blood routine, liver and kidney function, electrolytes, myocardial enzymes and cTNI (cardiac Troponin I, cTNI) were all normal. Electrophysiological examination indicated that sinus node function was normal. Informed writeen consent was obtained from the guardians, which was obtained in the consent to publish section as detailed in our editorial policies.

**Fig. 1:**
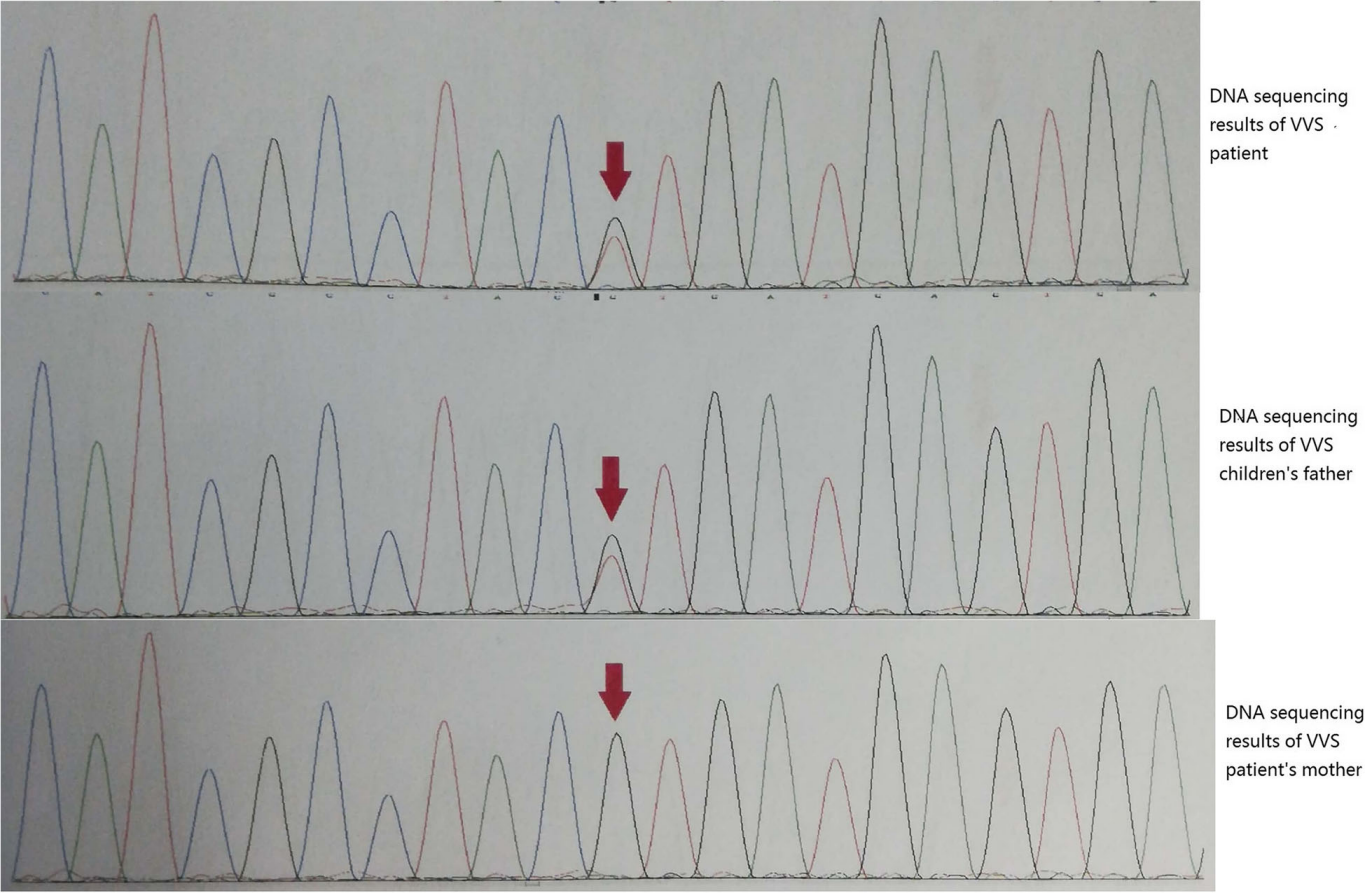
Children and their parents gene sequencing results: A heterozygous mutation in the exon coding region of the SCN5A gene, C. 5851G > T (coding region 5551 nucleotide changed from G to T), was detected in the peripheral blood of the patient. The mutation resulted in the change of amino acid 1951 from leucine (Val) to isoleucine (Leu), which may affect the function of the protein. The child’s father carries the mutation

A heterozygous mutation in the exon coding region of the SCN5A gene, C. 5851G > T (coding region 5551 nucleotide changed from G to T), was detected in the peripheral blood of the patient. The mutation resulted in the change of amino acid 1951 from leucine (Val) to isoleucine (Leu), which may affect the function of the protein. The child’s father carried the mutation (see Fig. 1). The variation is not a polymorphism, and the frequency of occurrence is very low in the population. The pathogenicity of the mutation has been reported in previous literature, and [8] was associated with Brugada syndrome. Sanger sequencing confirmed that the compound heterozygous mutation probably came from the father.

Combined with the clinical manifestations, test results, family history and gene mutation results, electrophysiological examination and modified vagal ganglion radiofrequency ablation were performed in the child. The ECG playback was normal on the second day after operation. Holter showed no abnormality and no chest tightness or syncope occurred after 3 months and one-year follow-up.

## Discussion and conclusions

SCN5A is a coding gene for sodium channel alpha-subunit. Previous studies have shown that SCN5A gene mutation is associated with long QT syndrome, Brugada syndrome and progressive familial heart block I [10–12]. However, the SCN5A gene mutation has not been reported in VVS and third-degree AVB, the mechanism for which remained unclear. Previous studies have suggested that the imbalance of autonomic nerve regulation, neurohumoral factors and abnormal cerebral blood flow regulation are potential factors involved in VVS pathogenesis [13]. Huang Y discovered that β1 adrenergic receptor gene also participated in the pathogenesis of VVS [14].

In the present case report, SCN5A gene mutation was observed in the VVS patient with third-degree AVB. According to an epidemiological survey in 1999, the prevalence of third-degree AVB in the United States was approximately 0.02%, and the global prevalence was nearly 0.04% [15, 16]. Previous studies showed that SCN5A gene mutation might be involved in the occurrence of third-degree AVB [4]. SCN5A is an alpha subunit (Nav1.5) that encodes the cardiac sodium channel and participates in the action of cardiac myocytes and the generation and transmission of bits. Cardiac natriuretic channels widely exist in atrial and ventricular myocytes and Purkinje fibers. In the 0 phase (depolarization phase) of action potential, sodium channels are opened up to produce an inward sodium. Ionic currents (I_Na_) form the ascending branch of action potential, which determines the excitability and conduction velocity of the heart. The ion channel is a glycosylated polypeptide complex consisting of a porous alpha subunit and four beta subunits. The alpha subunit is encoded by the SCN5A gene, including four homologous domains (DI-DIV). Each domain includes 6 trans-membrane segments (S1 - S6). SCN5A gene mutation contributed to sodium channel dysfunction which was associated with various inherited arrhythmias including long QT syndrome type third-degree, Brugada syndrome, cardiac conduction defect and etc [10–12] SCN5A gene mutation showed close relation with AVB. Deficient SCN5A gene mutation could decrease the function of sodium channel, decrease the I_Na_ at depolarization and depolarization velocity and peak value of cardiomyocytes during depolarization, and block the cardiac conduction system in varying degrees, eventually leading to the occurrence of AVB [4].

The patient developed syncope induced by car-sickness. Holter showed grade third-degree AVB. Peripheral blood gene test showed a heterozygous mutation c.5851G > T in SCN5A gene (coding region 5851 nucleotide changed from G to T), which resulted in the change of amino acid 1951 from Val to Leu (p.val1951leu) (Fig. 1), showing that SCN5A gene was extrinsic and heterozygous mutations existed in the coding region. Syncope occurrence, external hospital DC examination and HUTT results, combined with the clinical manifestations of the patient, diagnosis of VVS was made. However, the specific mechanism of SCN5A mutation in children with VVS combined with grade third-degree AVB needs to be further elucidated.

We retrieved literatures discovering that only one case report had described SCN5A gene mutation in a VVS child who was combined with Brugada syndrome [9]. The clinical manifestation of the patient was syncope episode. Medical examination performed when the patient was 8 years old revealed nonspecific intraventricular conduction delay and first-degree AVB and elevation of ST segment was not observed. Physical examination, chest x-ray film, echocardiography, and treadmill exercise testing were normal, and no ST elevation or arrhythmias were observed at that time. Because the syncopal attacks typically occurred while the patient was in an upright posture or was under emotional stress, his condition was diagnosed as mixed vasovagal syncope, although it was not proved at that time. Head-up tilt test provoked hypotension followed by 12 s of sinus arrest, indicating a mixed type I neurally mediated syncope. At age 17 years, a coved-type ST elevation was recorded from the third intercostal space and the diagnosis of Brugada syndrome was made. The patient had no family history of sudden cardiac death, but his mother had sick sinus syndrome with first-degree AV block, and his asymptomatic brother had first-degree AV block and nonspecific intraventricular conduction delay. An implantable cardioverter-defibrillator was recommended to the proband, but the patient declined. He has been treated with cilostazol, a phosphodiesterase inhibitor, to prevent severe bradycardia and possible arrhythmias due to Brugada syndrome. The two cases had common points as follows. Two patients were male, and the age of onset was adolescent. They were both referred for syncope, one of which was accompanied by chest tightness. The two cases both had family hereditary. The father of one child was attacked by VVS and the mother of the other had sinus first-degree AV block. However, two cases had differences. In our case report, a heterozygous mutation in the exon coding region of the SCN5A gene, C. 5851G > T (coding region 5551 nucleotide changed from G to T), was detected in the peripheral blood of the patient which resulted in the change of amino acid 1951 from leucine (Val) to isoleucine (Leu), and might affect the function of the protein. The child’s father carries the same mutation (see Fig. 1). Previous case revealed a novel SCN5A mutation at exon 2 resulting in a premature stop codon (Q55X) in the proband, his mother, and his brother.

Furthermore, a study demonstrated that 12 (35%) of 34 patients with a coved-type ST elevation showed a vasovagal response to head-up tilt test [17]. Moreover, based on the observation that SCN5A is ex-pressed not only in the myocardial cells but also in intra-cardiac ganglia, it is speculated that the nonsense mutation of SCN5A provides not only the substrate for Brugada syndrome in the myocardium but also an imbalance in intracardiac ganglia activity [18], which in turn results in autonomic dysfunction implicated in both Brugada syndrome and neutrally mediated syncope. These observations suggest an association between neutrally mediated syncope and third-degree AVB rather than a simple coincidence. Identification of the causes of syncope in such patients often is difficult; therefore, treatment of these patients remains a therapeutic challenge. Our report provides for the first time a genetic and biophysical basis that supports an association between neurally mediated syncope and third-degree AVB.

Up to now, treatment of VVS by drugs and pacemakers is not satisfying. A multi-center study suggested that the success rate of drug therapy and pacing in preventing recurrence of syncope was only 31.6–67% [19–23]. It was previously thought that pacemakers should be implanted in VVS patients with cardiac depression and third-degree AVB. However, pacemakers implantation had a risk of pacing system infection. In this study, the patient was treated with modified vagal ganglion ablation. After operation, the follow-up results achieved good results with no syncope and chest tightness symptoms recurrence. Yao et al. discovered that vagal ganglion ablation in ten VVS adults was effective in preventing syncope occurrence [24]. Pachon et al. suggested that catheter ablation for cardiac autonomic nervous system regulation was a feasible alternative therapy for refractory autonomic nerve-mediated syncope [25]. However, up to now, there is no report of modified vagal ablation for children with VVS combined with third-degree AVB. Our study speculated that modified vagal ablation had a good effect on children with VVS combined with third-degree AVB.

We demonstrated a novel nonsense SCN5A mutation in a VVS patient with third-degree AVB. The prognosis of vasovagal syncope might not necessarily be benign, because at least some patients with VVS, such as the present case, might also have third-degree AVB due to a subclinical genetic substrate that may give rise to lethal arrhythmias. Our findings expand the genotypic spectrum of this condition and provide a molecular basis for further studies of the mechanisms underlying SCN5A-associated in children with VVS.

## Supplementary information

**Additional file 1 (**JPG 4962 kb)

## Data Availability

All data generated during this study are included in this publication [and its supplementary information files]. No data analysis was provided during this study.

## Abbreviations

VVS: Vasovagal syncope
AVB: Atrioventricular block
HUTT: Head-up tilt test
